# An SNP Mutation of Gene *RsPP* Converts Petal Color From Purple to White in Radish (*Raphanus sativus* L.)

**DOI:** 10.3389/fpls.2021.643579

**Published:** 2021-06-03

**Authors:** Dongming Liu, Xiaochun Wei, Dongling Sun, Shuangjuan Yang, Henan Su, Zhiyong Wang, Yanyan Zhao, Lin Li, Jinfang Liang, Luming Yang, Xiaowei Zhang, Yuxiang Yuan

**Affiliations:** ^1^Institute of Horticulture, Henan Academy of Agricultural Sciences, Zhengzhou, China; ^2^College of Horticulture, Henan Agricultural University, Zhengzhou, China

**Keywords:** radish, transcriptome, anthocyanins, gene mapping, petal color

## Abstract

Along with being important pigments that determining the flower color in many plants, anthocyanins also perform crucial functions that attract pollinators and reduce abiotic stresses. Purple and white are two different colors of radish petals. In this study, two cDNA libraries constructed with purple and white petal plants were sequenced for transcriptome profiling. Transcriptome results implied that the expression level of the genes participating in the anthocyanin biosynthetic pathway was commonly higher in the purple petals than that in the white petals. In particular, two genes, F3′H and DFR, had a significantly higher expression pattern in the purple petals, suggesting the important roles these genes playing in radish petal coloration. BSA-seq aided-Next Generation Sequencing of two DNA pools revealed that the radish purple petal gene (*RsPP*) was located on chromosome 7. With additional genotyping of 617 F_2_ population plants, the *RsPP* was further confined within a region of 93.23 kb. Transcriptome and Sanger sequencing analysis further helped identify the target gene, *Rs392880*. *Rs392880* is a homologous gene to F3′H, a key gene in the anthocyanin biosynthetic pathway. These results will aid in elucidating the molecular mechanism of plant petal coloration and developing strategies to modify flower color through genetic transformation.

## Introduction

Anthocyanins are a group of glycosylated polyphenolic compounds widely present in plant tissues; they confer color to them, varying from orange, red, and purple, to blue. Not only do they play vital roles in controlling color expression, but these secondary metabolites also possess some crucial functions in reducing damage from drought stress, cold, UV irradiation, and microbial agents in plant tissues (Christie et al., 1994; Sarma and Sharma, 1999; Lorenc-Kukuła et al., 2005; Castellarin et al., 2007). For example, to protect the plants from environmental stress, anthocyanins may help fight pathogens or act as UV screens and antioxidants *via* accumulation in specialized cells (Treutter, 2005). One of their most essential functions is influencing petal coloration, which is integral to the successful attraction of pollinators and seed distributors (McCall et al., 2013; Veiga et al., 2015). The primary background petal color in plants is firstly determined by the content and ratio of three kinds of anthocyanidins: pelargonidin determines the orange to brick red colors, delphinidin determines the purple to blue colors, and cyanidin determines the red to pink to blue colors. Secondly, petal coloration varies with changes in PH and structural modifications of the anthocyanidins (Tanaka et al., 1998).

Anthocyanins belong to a diverse family of metabolites called flavonoids that contains six members: anthocyanins, chalcones, flavones, flavonols, flavandiols/proanthocyanidins, and aurones. To ascertain the importance of anthocyanins to plants, as well as to humans, extensive research has been performed to elucidate the metabolic pathway of anthocyanins in plants during the past decades (You et al., 2019; Zhang D. W. et al., 2020). Anthocyanins are synthesized initially from phenylalanine by several enzymes, including phenylalanine ammonia-lyase (*PAL*), cinnamic 4-hydroxylase (*C4H*), and cinnamic 4-coumarate-CoA ligase (*4CL*). Then, the tetrahydroxychalcone (THC) is catalyzed by the chalconesynthase (*CHS*) from one 4-coumaroyl-CoA and three malonyl-CoA. The synthesized THC is then isomerized to the (2S)-naringenin *via* catalyzation of chalcone isomerase (*CHI*). With hydroxylation at the 3-position of (2S)-naringenin by flavanone 3-hydroxylase (*F3H*), (2R,3R)-dihydrokaempferol is obtained. (2R,3R)-dihydrokaempferol is further hydroxylated by flavonoid 3′-hydroxylase (*F3′H*) or flavonoid 3′,5′-hydroxylase (*F3*′*5*′*H*) into two other dihydroflavonols: dihydroquercetin or dihydrotricetin, respectively. Hydroxylation with F3′H or F3′5 ′H is closely related to the hydroxylation pattern of the B-ring of flavonoids and anthocyanins. Next, all obtained dihydroflavonols are reduced to their corresponding colorless leucoanthocyanidins by dihydroflavonol 4-reductase (*DFR*) and then be converted to colorful anthocyanidins by anthocyanidin synthase (*ANS*). Finally, stable anthocyanidins are formed from the synthesized anthocyanidins encoded by UDP-glucose: flavonoid 3-O-glucosyltransferase (*UFGT*). Along with the necessary structural genes, a couple of transcription factors, including R2R3-MYB, MYB114, bHLH, WD40, WRKY, and NAC (Zhou et al., 2015; Xie et al., 2016; Lloyd et al., 2017; Yao et al., 2017; Liu et al., 2018; An et al., 2019), have been reported to be linked with anthocyanins formation by affecting the expression of the structural genes or through a complex regulatory network.

As a root vegetable and a relative of *Brassica rapa* and *Brassica oleracea* plants, radishes (*Raphanus sativus* L., 2n = 2x = 18) are sources of fiber, vitamins, mineral elements, and health-promoting nutrients, and they are cultivated worldwide (Zieliñski et al., 2005; Siddiq and Younus, 2018). Except for the petals, the color of the radish flesh, stem, and leaf in some cultivars is also purple or red due to the presence of anthocyanins. For example, the Red-fleshed radish (*Raphanus sativus* L.) is a unique cultivar whose taproot is rich in anthocyanins, but a CACTA transposon-induced methylation of the promoter of gene *RsMYB1* was proposed to be responsible for the white-fleshed mutant (Wang et al., 2020). Gene *RsMYB90* was found to be a key gene determining anthocyanins accumulation and taproot skin color (Luo et al., 2020). Besides the genes, some microRNAs concerned with anthocyanin biosynthesis were also identified by transcriptome analysis (Sun et al., 2017). In the radish petals, cyanidin 3-O-[2-O-(2-O-(*trans*-caffeoyl)-b-glucopyranosyl)-6-O-(*trans*-p-coumaroyl)-b-glucopyranoside]-5-O-[6-O-(malonyl)-b-glucopyranoside] and cyanidin 3-O-[2-O-(2-O-(*trans*-caffeoyl)-b-glucopyranosyl)-6-O-(*trans*-feruloyl)-b-glucopyranoside]-5-O-[6-O-(malonyl)-b -glucopyranoside] were found to be the major floral anthocyanins (Tatsuzawa, 2016). But the inheritance pattern and genes of radish petal color are still not reported. Because of the vital performance of heterosis, most of the available radish cultivars are hybrid cultivars now. For the considerable contribution Ogura cytoplasmic male sterility (CMS) made to hybrid seed production in radishes, the role of petal color is stressed for the possible effect of petal color on attracting pollinators (Sutherland and Vickery, 1993). In this study, comprehensive transcriptome analysis and functional characterization of the DEGs were completed. In addition, with genome resequencing of two DNA pools from the F_2_ population, the *RsPP* (Radish Purple Petal) gene was confined to a candidate region of 93.23 kb on chromosome 7, and gene *Rs392880* was identified as the target gene for the *RsPP*. These results provide new insight into the molecular mechanism of radish petal color formation and aid in elucidating pigment study in radish.

## Materials and Methods

### Plant Materials

**FIGURE 1:**
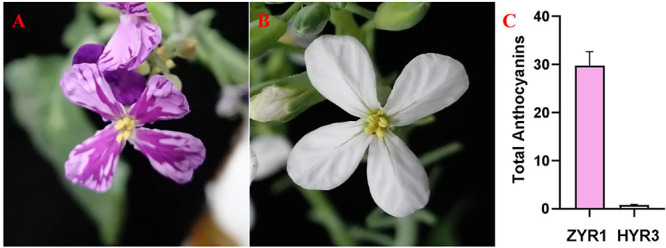
Phenotypes of purple and white petals and relative anthocyanins content in ZYR1 and HYR3. **(A)** Purple petal phenotype of cultivar, ZYR1. **(B)** White petal phenotype of cultivar HYR3. **(C)** Relative anthocyanin content in the petals (mg/g). Error bars indicate standard errors (SEs).

Two representative radish cultivars with phenotypes purple (ZYR1) and white petals (HYR3) were selected (Figure 1). Both of the radish cultivars were grown in a greenhouse with the same growth conditions and environments. The purple and white petal phenotypes were visually observed and recorded when they could be easily distinguished. Total anthocyanin was extracted after flowers bloomed in the morning and determined following the steps outlined in a previous study (Barth et al., 2010). ZYR1 was taken as the female plant used to cross with HYR3 to generate F_1_ and F_2_, BC_1_P_1_, and BC_1_P_2_ populations for inheritance analysis and gene mapping. Segregation ratios of purple/white petals in the F_2_ population were analyzed with Chi-square tests (χ^2^).

### BSA-seq Analysis and Mapping of Gene *RsPP*

Total genomic DNA in parental, F_1_, and 617 F_2_ population plants was extracted from young leaves *via* the cetyltrimethylammonium bromide (CTAB) method, and the concentration was adjusted to 80 ng/uL (Saghai-Maroof et al., 1984). To obtain the candidate genomic region related to the radish petal color, two DNA pools named P-pool and W-pool were constructed. P-pool was obtained by mixing equal amounts of DNA from 30 purple-petal plants and W-pool was obtained by mixing equal amounts of DNA from 30 white-petal plants. The two DNA pools were sequenced on an Illumina HiSeqTM 2500 platform. After low-quality and short reads were filtered out with FastQC (Andrews, 2010), the remaining high-quality reads of each pool were mapped onto the radish reference genome sequence^1^ by BWA (Li and Durbin, 2009). Single-nucleotide polymorphism (SNP) calling followed using GATK Best-Practices (McKenna et al., 2010). High-quality SNPs were used for Bulked-Segregant (BSA-seq) analysis and the Euclidean Distance (ED) algorithm (Li and Durbin, 2009); they were then used to identify the regions associated with purple petals in the radish. The calculation of ED was completed using MMAPPR (Mutation Mapping Analysis Pipeline for Pooled RNA-seq) (Hill et al., 2013) and the high ED value suggested that the SNPs in the genomic regions were closely associated with the targeted genes associated with purple petal in radish (Hill et al., 2013).

### Fing Mapping of Gene *RsPP*

To validate the BSA-seq results and further narrow down the region containing the target gene, 50 pairs of Indel primers were developed according to the comparative genomic information of the P-pool and W-pool (Supplementary Table 1). The primers showing sufficient polymorphism were further used to genotype the F_2_ population plants. PCR amplification of molecular markers and gel electrophoresis were conducted as described previously (Liu et al., 2017). Sequences of primers used for mapping are listed in Supplementary Table 1.

### Identification and Sequence Analysis of the Candidate Gene

The expression pattern of the candidate gene in ZYR1 and HYR3 was tested using RT-qPCR with ABI SYBR green on an ABI 7900HT Fast Real-Time PCR System (Applied Biosystems) following the manufacturer’s instructions. The primers used for qPCR were listed in Supplementary Material and β*-actin* gene was used as the reference gene. Each sample was tested in triplicate. The BLAST program^2^ (Aron et al., 2011) was employed to analyze the genes within the mapping region. Sequences were aligned with the software MultAlin^3^ (Florence, 1988). The gene structure was analyzed with the program FGENESH (Solovyev et al., 2006).

### Transcriptome Library Construction

Petals of the two cultivars were collected at the same time when flowers began to bloom. Three frozen petals from three different plants (numbered P1 to P3/W1 to w3) were randomly selected for RNA extraction in each replicate. Total RNA was extracted with the EasyPure^®^ Plant RNA Kit (TransGen Biotech Co., Ltd.) following the manufacturer’s instruction and DNA was removed with RNase-free DNase. After concentration and quality of RNA were detected, mRNA was fragmented into small pieces, and first-strand cDNA was synthesized with a random hexamer primer and M-MuLV Reverse Transcriptase. Then the second-strand cDNA was synthesized with DNA Polymerase I and RNase H, and the remaining overhangs were then converted into blunt ends. Finally, PCR was performed with Phusion High-Fidelity DNA polymerase and universal PCR primers, Index (X) Primer, and PCR products were purified by the fdAMPure XP system.

### Transcriptome Data Analysis

To investigate the mechanisms corresponding with the anthocyanin accumulation and petal coloration, six cDNA libraries were constructed with petals of ZYR1 and HYR3 and subjected to RNA-seq analysis based on an Illumina HiSeq 2000 platform by Personal Biotechnology Co., Ltd. (Shanghai, China). The raw data were deposited in the National Center for Biotechnology Information (NCBI) with the accession number PRJNA549842. After the low-quality reads and the reads containing adapter and ploy-N sequence were removed from the raw reads, clean reads were obtained and were further aligned to radish reference genome sequences released by the Radish Genome Database^4^ using TopHat 2.0.12 (Trapnell et al., 2009). The mapped reads count was normalized with FPKM (Fragments-per-kilobase of transcript per-million-fragments mapped) (| log_2_^*FoldChange*^| > 1, *P*_value < 0.05) to provide a gene expression level estimation.

Expression analysis among samples was calculated using the DESeq R package. The significant P-value between samples was determined using the Benjamini and Hochberg method (Benjamini and Hochberg, 2000). DEGs (differentially expressed genes) were obtained using a DESeq2 program with an adjusted *P* value less than 0.05 and | log2FoldChange| > 1 based on the FPKM values (Love et al., 2014). A Gene Ontology (GO) enrichment analysis of the DEGs was implemented using the GOseq R package with a corrected *P* < 0.05 (Young et al., 2012). KOBAS (KEGG Orthology Based Annotation System) software was employed to identify the enriched pathways of DEGs based on the KEGG database (Kanehisa and Goto, 2000).

Real-time quantitative PCR (RT-qPCR) was used to verify the data from the transcriptome. RT-qPCR was carried out with ABI SYBR green on an ABI 7900HT Fast Real-Time PCR System (Applied Biosystems) following the manufacturer’s instructions. β*-actin* gene was used as the reference gene. The reaction parameters were carried out following a previous research paper (Liu et al., 2020) and the relative expression levels were evaluated using the 2 ^–Δ^ ^Δ^ ^*Ct*^ method (Livak and Schmittgen, 2001). All reactions were performed using three technical replicates. Sequences of primers for RT-qPCR are listed in Supplementary Table 1.

## Results

### Phenotype and Anthocyanin Content

Visual inspection of the petals showed that, in contrast to the consistent coloring of the white petals of HYR3 (Figure 1B), all the flower petals of ZYR1 plants exhibited a purple appearance (Figure 1A), and the color depth varies in ZYR1 petals (Figure 1A). The total anthocyanin content of the ZYR1 petals was significantly higher than that of HYR3 petals, although some anthocyanin accumulation was observed in HYR3 (Figure 1C). This was in accordance with the expected accumulation based on the colors of the flowers. These results indicated that the drastic differences in anthocyanin accumulation were a result of genetic specificity between different cultivars.

### Mapping of *RsPP* Gene Based on BSA-seq Analysis

The purple petal phenotype in the F_2_ population was easily identified after blooming, and a total of 617 F_2_ population plants were observed. Among the F_2_ plants, 468 displayed purple petals, and 149 displayed white petals, which was consistent with a 3 to 1 segregation ratio (*P* = 0.24 in a χ^2^ test against 3:1). In the BC_1_P_1_ population, which was obtained through backcross of F_1_ plants with HYR3, the number of purple-petals plants and white-petals plants is 98 and 106, displaying a ratio of 1:1 (*P* = 0.33 in a χ^2^ test against 1:1). Furthermore, petals of all BC_1_P_2_ plants, which were achieved through the backcross of F_1_ plants to ZYR1, are purple. These results indicated that the purple petal trait in radish follows a single-dominant inheritance pattern.

**FIGURE 2:**
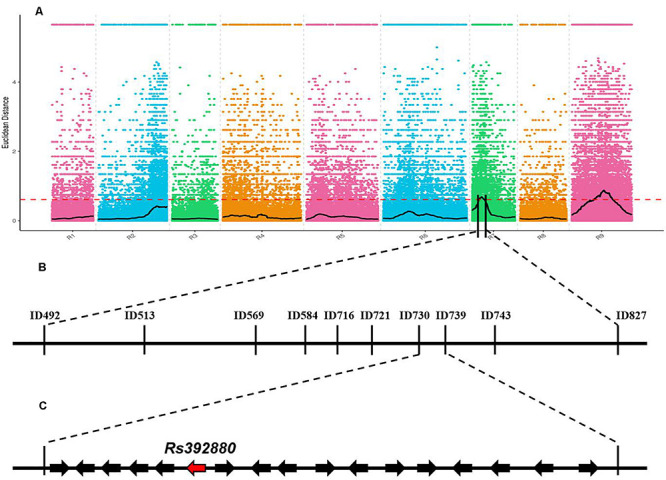
BSA-seq based mapping of gene *RsPP* BSA-seq analysis using Euclidean Distance (ED) algorithm **(A)**. The candidate gene was confined to a region of 93.23 kb on chromosome 7 **(B,C)**. The red dashed line represents the significant threshold.

After low-quality reads were removed from the two bulks, a total of 10.64 Gb clean data were obtained (P-bulk, 5.23 Gb; W-bulk, 5.41 Gb) with an average depth of 15 × the genome assembly. After SNPs with low coverage and discrepancy between the two bulks were filtered, a total of 1,343,573 high-quality SNPs and 519,983 Indels were identified. To obtain the genomic region associated with the purple petal phenotype, the ED algorithm was used to calculate the allele segregation of SNPs between the two bulks. In the ED algorithmic analysis, there were two significant regions identified that could be associated with the purple petal trait, located in 4.32 to 7.49 Mb of chromosome 7 and 11.58 to 14.77 Mb of chromosome 9 (Figure 2A), implying that there existing two loci are responsible for the petal color. The BSA-seq result was inconsistent with the inheritance analysis. To screen for the correct region containing gene *RsPP*, six pairs of Indel markers from these two predicted regions were developed according to the comparative genomic information of the two DNA pools. After polymorphism screening by the two parental lines, four Indel primers (two from chromosome 7 and two from chromosome 9) showed clear bands and adequate polymorphism, and they were then used for genotyping the F_2_ segregating population containing 617 plants. As a result, both the markers from chromosome 7 revealed a close genetic linkage with the petal color trait, but the primers from chromosome 9 were not genetically linked with it, implying that the target gene for purple petals was located on chromosome 7 but not chromosome 9. To further isolate the target gene, 30 pairs of Indel primers in the predicted region on chromosome 7 were developed and used to genotype the F_2_ plants. Subsequently, the *RsPP* gene was confined to a region covering a physical distance of 93.23 kb (from 7,302,375 to 7,395,600) (Figure 2C). According to the radish reference genome information, a total of 17 genes were located in the mapped region (Figure 2C).

### Candidate Gene Identification

**FIGURE 3:**
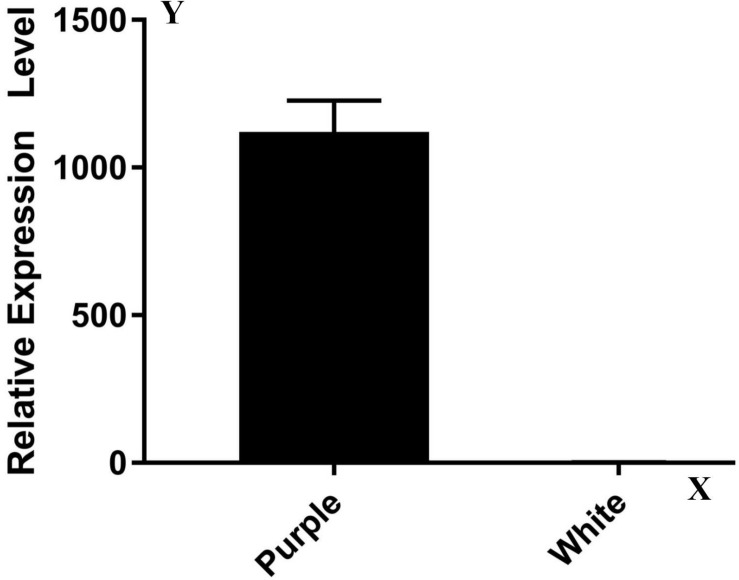
Expression levels of genes *Rs392880* in ZYR1 and HYR3. The x-axis displays different cultivars. The y-axis displays the relative transcription level of the *Rs392880* gene compared with actin.

A transcriptome analysis result showed that most of the 17 genes (except *Rs392880*) owned a similar expression pattern between ZYR1 and HYR3 (Supplementary Table 2). The expression levels of the gene *Rs392880* in the cultivar HYR3 were significantly down-regulated compared with that of ZYR1. This was confirmed by the qPCR result (Figure 3). Gene function analysis implied that *Rs392880* codes for the flavonoid 3′-hydroxylase and a conserved domain belonging to the P450 superfamily, indicating that *Rs392880* is a homologous gene for F3′H. Except for gene *Rs392880*, gene function analysis showed that the remaining 16 genes in the mapping region were irrelevant with anthocyanins metabolism (Supplementary Table 3). The gene expression data and functional analysis result indicated that the *Rs392880* should be the target gene responsible for the petal color in radish.

**FIGURE 4:**
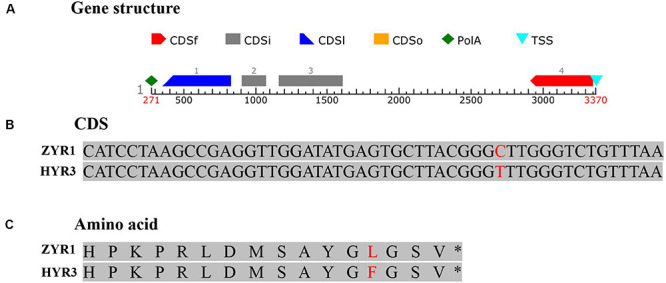
The gene structure and mutational pattern of gene *Rs392880* in HYR3. **(A)** The structure of gene *Rs392880*. **(B)** The SNP in gene *Rs392880*. **(C)** The mutational pattern in the level of amino acids of *Rs392880*.

To substantiate this result, genomic DNA of gene *Rs392880* in the parental materials was sequenced. Four exons existed in gene *Rs392880* (Figure 4A). Except for SNP (G to A, base site of 7,319,959 on chromosome 7) in the fourth exon (Figure 4B), no base variance was found of gene *Rs392880* between ZYR1 and HYR3. The SNP changed leucine to phenylalanine in the amino acid sequence (Figure 4C). A pair of primers was developed according to the identified SNP in gene *Rs392880* to verify the consistency between the SNP and petal color phenotype (Supplementary Table 1). Among the F_2_ plants, 100 individuals including all recombinants were used to check the polymorphism. The result showed that 26 purple-petal individuals were homozygous dominant and 50 were heterozygous, whereas 24 white-petal individuals were homozygous recessive, just consistent with the petal color phenotype. These results suggested that gene *Rs392880* should be the key gene responsible for the purple petals in radish.

### Functional Annotation and Classification of the DEGs

**FIGURE 5:**
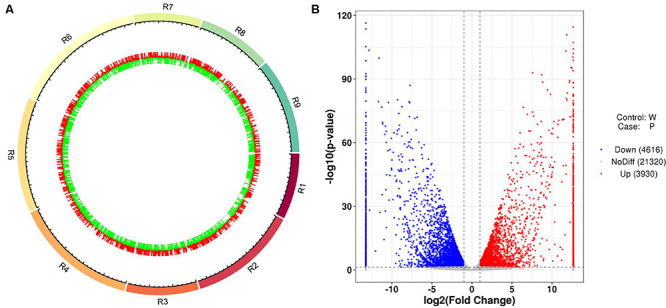
Differentially expressed genes (DEGs) obtained between ZYR1 and HYR3 by the transcriptome analysis. **(A)** DEGs within the radish chromosomes. Red and green columns indicate the upregulated and downregulated genes in ZYR1 vs HYR3. **(B)** Volcano plot of the DEGs in ZYR1 vs HYR3. The red and blue dots indicated significantly upregulated or downregulated expression of genes in ZYR1 compared to HYR3.

After the unreliable reads were removed, clean reads of high quality were obtained, and the sequencing and assembly results demonstrated high reliability for further analysis (Table 1). The correlation coefficients of the six samples were also analyzed and are listed in Supplementary Table 4. Based on the FPKM values, a total of 8,546 DEGs between ZYR1 and HYR3 were obtained (Supplementary Table 5). All the DEGs were evenly distributed over the nine radish chromosomes (Figure 5A). Compared with HYR3, 3930 genes were up-regulated and 4,614 were down-regulated in ZYR1 (Figure 5B).

**TABLE 1:** Statistical summary of the transcriptome assembly for ZYR1 and HYR3.

**Sample**	**P1**	**P2**	**P3**	**W1**	**W2**	**W3**
Clean Reads No.	47,275,642	39,625,232	39,933,998	42,509,034	46,460,884	39,644,726
Clean Reads%	92.43	92.05	92.5	91.66	92.24	92.2
Clean Data (bp)	7,138,621,942	5,983,410,032	6,030,033,698	6,418,864,134	7,015,593,484	5,986,353,626
Clean Data%	92.43	92.05	92.5	91.66	92.24	92.2
Q20 (%)	97.97	97.93	98.03	97.53	97.97	97.54
Q30 (%)	94.91	94.84	95.03	94.03	94.91	94.09
mapped to genome	38,718,583 (81.90%)	32,280,868 (81.47%)	32,361,406 (81.04%)	36,696,053 (86.33%)	40,959,132 (88.16%)	32,341,575 (81.58%)

*W1, W2, and W3 are the three samples from HYR3, and P1, P2, and P3 are the three samples from ZYR1.*

**FIGURE 6:**
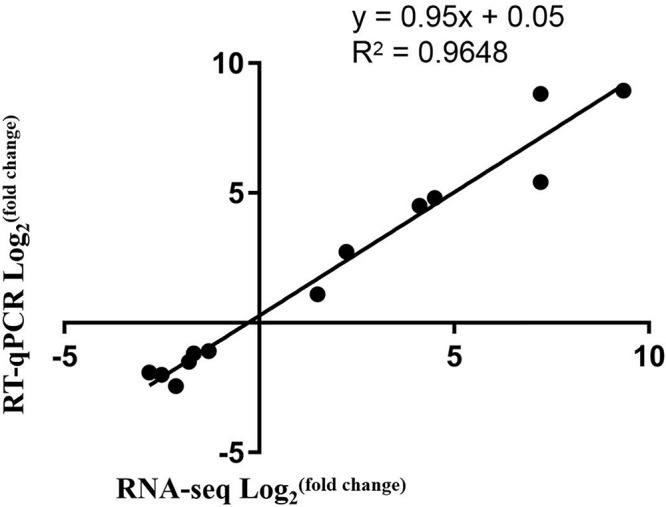
Correlation of expression levels between RNA-Seq and qRT-PCR.

To validate the RNA-Seq data, qRT-PCR for 13 DEGs was conducted. The selected 13 DEGs were related to anthocyanin biosynthesis. Comparison of the qRT-PCR and the RNA-Seq data showed that trends of the gene expression patterns were consistent and had a strong positive correlation coefficient (Figure 6), indicating that the RNA-Seq data was reliable.

**FIGURE 7:**
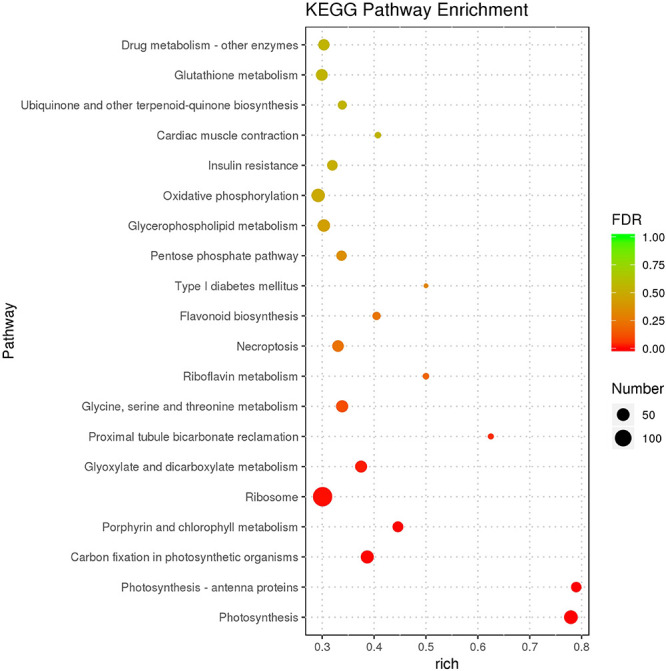
Scatter plot of the KEGG pathway enrichment of DEGs. The size of the dots represents the number of genes, and the color of the dots represents the range of the q-value.

**FIGURE 8:**
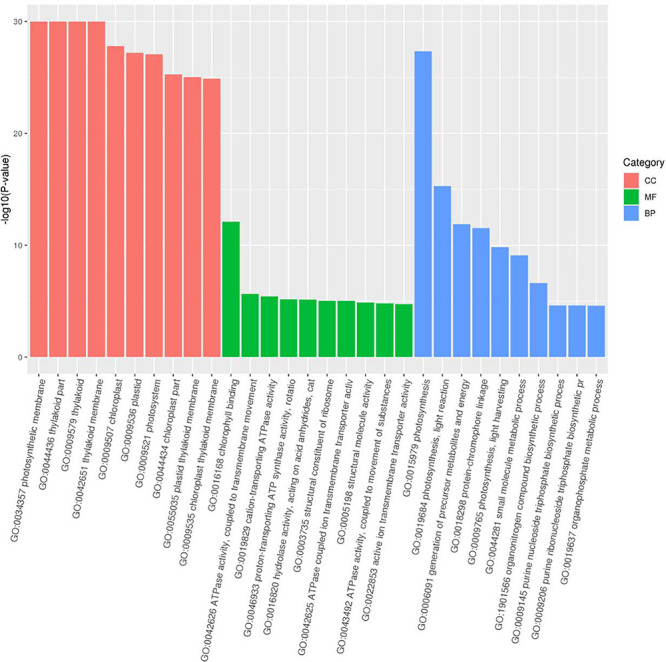
Gene Ontology (GO) functional classification of DEGs.

As the top enriched KEGG pathways, shown in Figure 7, the pathways concerning flavonoid biosynthesis, glutathione metabolism, photosynthetic antenna proteins, and photosynthesis were enriched. The pathway enriched with DEGs may be the key reason for the varied phenotypes or the result of another enriched pathway (Figure 7). To classify the function of the DEGs, Gene Ontology (GO) enrichment was carried out (Figure 8). In the “Biological Process,” “Cellular Component,” and “Molecular Function” categories, plenty of genes related to the flavonoid and pigmentation metabolic processes were obtained. The top enriched GO terms in the cellular component category were photosynthetic membrane and thylakoid-related terms (Figure 8). For the biological process category, the most enriched terms were photosynthesis, light reaction, and generation of precursor metabolites and energy, implying a close relationship between the anthocyanin and photosynthesis biological metabolisms (Figure 5). The top terms in the molecular function category were chlorophyll-binding, ATPase activity, and cation-transporting ATPase activity, which are related to the energy processes for different anthocyanin metabolisms (Figure 8).

### Expression Analysis of Putative Genes Involved in Anthocyanin Biosynthesis

**FIGURE 9:**
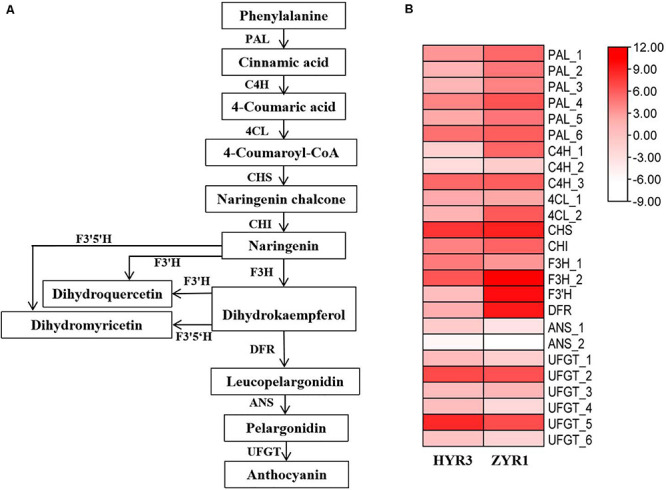
Gene expression profiles related to the anthocyanin biosynthesis. **(A)** The putative anthocyanin biosynthetic pathways in plants. **(B)** Heatmap of genes related to anthocyanins metabolism. Scale bars show log 2 -transformed FPKM values of each gene. The intensity of colors indicates the expression levels of the genes.

Based on the KEGG database analysis, 25 anthocyanin biosynthesis-related genes were identified (Figure 9), including six *PAL* and three *C4H* syntenic genes, one for each of the *CHS*, *CHI*, *F3*′*H*, and *DRF* genes; two genes for each of *4CL*, *F3H*, and *ANS*, and six genes for *UFGT* (Figure 9A). However, the gene for *F3*′*5*′*H* was not identified in the radish anthocyanin biosynthetic pathway. According to the transcriptome result, most of the genes associated with anthocyanin biosynthesis in ZYR1 are commonly more highly enriched than in *HYR3* (Figure 9B). For example, the expression level of genes *PAL_1*, C*4H*_*1*, *4CL_2*, *CHI*, *F3H_2*, in ZYR1 is higher than that in HYR3. Except for the above genes, the F3′H and DFR expression was significantly up-regulated in ZYR1 (Figure 9B). Different from the above genes, the expression of *F3H_1*, *AN*S_1, and some *UFGT* genes (*UFGT_1*, *UFGT_4*, *UFGT_5*, and *UFGT_6*) was down-regulated in ZYR1 (Figure 9B). All results suggested that the different expression patterns of these genes may be related to the coloration difference in cultivars HYR3 and ZYR1.

## Discussion

Plant coloration has always been a hotspot in plant biological research. The biosynthetic pathways of anthocyanins have been extensively characterized in higher plants such as in *Arabidopsis thaliana* (Lepiniec et al., 2006), peach (Zhao et al., 2020), carrot (Curaba et al., 2020), and potato (Zhang H. et al., 2020). Anthocyanins, carotenoids, and betalains are the basic primary pigments that determine plant color (Grotewold, 2006). Anthocyanins commonly confer the plant with orange, pink, red, purple, blue, and blue-black (Tanaka et al., 2008; Davies et al., 2010), and the type and content of the anthocyanin are believed to be the key in determining flower coloration (Kumar and Yadav, 2013; Dasgupta et al., 2017). In ZYR1 and HYR3, we found a dramatic reduction of anthocyanin content, implying a close relationship between the petal color appearance and pigment content changes (Figure 1C).

Anthocyanin biosynthetic pathways have been extensively characterized in higher plants. The key genes of metabolic pathways during anthocyanin biosynthesis were studied with RNA-Seq technology to explore the transcriptomic differences between two radish cultivars. The expression levels of nine genes in the pathway were up-regulated or down-regulated (Figure 9), indicating that the different color appearance was closely related to the varying gene expression. In the anthocyanin biosynthetic pathway, *trans*-cinnamic acid is initially formed through the deamination of phenylalanine by phenylalanine ammonia lyase (*PAL*). Then cinnamoyl-CoA and *p*-coumaroyl-CoA would be produced catalyzed by 4-coumarate-CoA ligase (4CL) and *trans*-cinnamate 4-monooxygenase (C4H) (Vogt, 2010). The up-regulated expression of PAL_1, C4H_1, and 4CL_2 in ZYR1 implying that these genes were more positively expressed in the purple petals and are deduced to be concerning with the different coloration in ZYR1 and HYR3. The formed *p*-coumaroyl-CoA is then isomerized to be flavanone catalyzed by chalcone synthase (CHS) and chalcone isomerase (CHI) (Petroni and Tonelli, 2011). The formed flavanones are catalyzed by the enzyme flavanone 3-hydroxylase (F3H) to dihydroflavonols. According to the transcriptome analysis result, the expression of genes CHI and F3H_2 were up-regulated but gene F3H_1 was down-regulated in ZYR1. The different expression patterns of F3H_1 and F3H_2 imply that these two genes may play different roles during anthocyanin biosynthesis. Dihydroflavonols, through flavonoid 3′, 5′-hydroxylase (F3′5′H), flavonoid 3′-monooxygenase (F3′H), dihydroflavonol 4-reductase (DFR), anthocyanidin synthase (ANS), and flavonoid-O-glycosyl-transferase (UFGT), catalyzes the final formation of pelargonidin, cyanidin, and delphinidin, involved in anthocyanin biosynthesis. The significantly up-regulated expression pattern of F3′H and DFR in ZYR1 implying the close relationship between these two genes and the different radish petals colors. The gene mapping result confirmed the supposition that F3′H could be the key gene for the color varying in ZYR1 and HYR3 (Figure 4). Inconsistent with the other pathway genes, the UFGT genes were commonly down-regulated in ZYR1, the implicated mechanism for the unusual expression pattern needs to be further study.

In radish, some types of anthocyanins have been identified and were reported to be responsible for red/purple root peels and petals (Kato et al., 2013; Tatsuzawa, 2016), but the genes related to petal color were still not isolated. In the present study, two regions were supposed to be associated with the purple petal trait by the BSA-seq analysis (Figure 2A), but the linkage analysis result proved that *RsPP* was located on a region on chromosome 7 (Figure 2B). It was deduced that the region on chromosome 9 was wrongly predicted because another trait was analyzed with these two DNA pools at the same time, and the peak on chromosome 9 may therefore be linked with the gene for another trait.

The gene *Rs392880* codes for a domain belonging to the cytochromes P450 protein superfamily protein is speculated to be responsible for the purple petal. As one of the largest protein families identified in plants, animals, fungi, bacteria, and viruses (Lamb et al., 2009). the P450 protein superfamily proteins play roles in many metabolic pathways since they can produce crucial secondary metabolites, including flavonoids, anthocyanins, isoflavones, and terpenoids (Hallahan and West, 1995; Schoenbohm et al., 2000). Cytochromes P450 is vital to the biosynthesis of flavonoids and anthocyanins, both of which are pigments responsible for determining major flower coloration. Flavonoid 3′-hydroxylase (F3′H) and flavonoid 3′,5′-hydroxylase (F3′5 ′H), two members of the cytochromes P450 superfamily, are the two key factors determining the number of hydroxyl groups on compounds by specifying and catalyzing hydroxylation of flavanones, dihydroflavonols, flavonols, and flavones. Flavanones and dihydroflavonols are the two enzymes that determine the hydroxylation pattern of these compounds (Tanaka and Brugliera, 2013), which significantly influences anthocyanin color. When expression of *F3*′*5*′*H* and/or *F3*′*H* genes is suppressed and a correctly identified DFR gene was over expressed, the biosynthetic pathway for anthocyanidin changes to pelargonidin biosynthesis, and an intense red color is yielded, thereby illustrating that the *F3*′*5* ′*H* and *F3*′*H* genes are powerful molecular tools for flower color modification (Tanaka et al., 2009). In the present study, although the *F3*′*H* gene expression level was down-regulated, petal color in cultivar HYR3 turned to be white but not intense red, the reason was supposed that the expression of the DFR gene was not up-regulated (Figure 9B).

With the help of a couple of flower color mutants in many plant species, various isoforms of the *F3*′*H* genes have been isolated and functionally characterized, such as those in *A. thaliana* (Han et al., 2010; Rao et al., 2020), barley (Himi and Taketa, 2015), potato (Jung et al., 2009), and many others. In the present study, using two radish cultivars with different petal colors, the gene *Rs392880* coding for flavonoid 3′-hydroxylase was identified and predicted to be the target gene determining the purple petal phenotype. Consistent with the previous studies, there was a loss of function mutation of the gene *Rs392880* due to a nucleotide change results in a switch from purple petals to white, implying that the gene *Rs392880* in radish also participates in anthocyanin metabolism. These results will help us to further understand the color variance in radish and help us develop strategies to modify flower color through genetic transformation.

## Data Availability Statement

The original contributions presented in the study are publicly available. This data can be found here: NCBI, accession number PRJNA685623.

## Author Contributions

DL, XW, LY, YY, and XZ designed the study. DS and SY performed the RNA isolation and qRT-PCR experiments. DL, XW, and HS performed the data analysis. DS, ZW, YZ, LL, and JL participated in the gene mapping. LY, YY, and DL wrote and revised the manuscript. All authors read and approved the final version of this manuscript.

## Conflict of Interest

The authors declare that the research was conducted in the absence of any commercial or financial relationships that could be construed as a potential conflict of interest.

## Footnotes

1 http://radish-genome.org/

2 http://www.ncbi.nlm.nih.gov/Structure/cdd/wrpsb.cgi

3 http://multalin.toulouse.inra.fr/multalin/multalin.html

4 http://radish-genome.org/Data_resource/index.php

## Supplementary Material

Supplementary Table 1 Primers used in this study.

Supplementary Table 2 Transcriptomic data of the genes within the mapping region.

Supplementary Table 3 Function analysis of the genes within the mapping region.

Supplementary Table 4 PCA of transcriptomic dataset analysis.

Supplementary Table 5 DEGs between ZYR1 and HYR3 with transcriptome analysis.
